# The Diagnosis of the Internal Secretory Disorders

**Published:** 1916-07

**Authors:** Henry R. Harrower

**Affiliations:** M. D., F. R. S. M. (Lond.), Los Angeles, California


THE
### TEXAS MEDICAL JOURNAL
**Dr. F. E. Daniel, Founder. Established July, 1885**

MRS. F. E. DANIEL, - Publisher and Managing Editor

**Published Monthly.—Subscription, $1.00 a Year.**

VOL. XXXII AUSTIN, JULY, 1916 No. 1

**The publisher is not responsible for views of contributors**

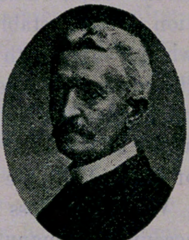

### Original Articles.

### The Diagnosis of the Internal Secretory Disorders.

BY HENRY R. HARROWER, M. D., F. R. S. M. (LOND.), LOS ANGELES, CALIFORNIA.

### III.

### THE DETECTION OF THE MINOR THYROID DYSCRASIAS—CONTINUED.

From what has been said regarding the exciting and predisposing cause of thyroid disorder, it will be clear that the history, both personal and family, is particularly important, as in it we may find a strong hint as to the prospective presence of the condition for which we are looking, although we may not always find a basic reason of this character in our anamnesis, for thyroid disturbances have a habit of appearing without the necessary hereditary or even the presumably essential etiologic foundation.

Hypothyroidism may be found at any time during life, from infancy to old age, though it is most common in young persons. The more serious forms are likely to show themselves in infancy or youth, while the forms that are usually overlooked altogether are more usual during the thirty or more years of active reproductive life and, as has been mentioned, it is especially frequent in women.

When we recall the principal intra-cellular functions of the thyroid hormone, it will be easy to understand that aberrations in the production of this chemical messenger not only interfere with cellular growth, but they derange the essential chemical changes connected with the incessant regeneration of the cells themselves. Their waste products are retained and the effete material is not burned up, facts which are proved in several different ways. The chief result of this special form of suboxidation is the establishment of a condition of *cellular infiltration* which varies both in degree and in the number and location of the organs attacked. That it is often generalized cannot be denied, but that it is more manifest in some tissues is also true, as we as see shall see when the results of the more serious forms of thyroid disease are considered.

While the loss of the normal thyroid hormone stimuli may account for many disorders, more clinical symptoms result from this infiltration than from any other single result of thyroid derangement. As we enumerate the symptoms of thyroid disorder, this one factor—infiltration—stands out above all the others, and when its importance and extent, as well as the fundamental philosophy of its presence, is thoroughly understood, it will explain many a symptom which previously had not been supposed to have the least to do with this gland. The credit for the discovery and announcement of this phenomenon undoubtedly belongs to my good friend Dr. Eugene Hertoghe, of Antwerp; and to him is due the homage of the medical profession for his remarkable contributions of this subject. The importance of the whole subject may be impressed by a quotation from this eminent authority:* "It is obvious that myxedematous dwarfism and infantile cretinism cannot escape detection by a physician of even moderate attainments, * * * but the slighter forms of thyroid inadequacy are almost invariably missed; yet, owing to their extreme prevalence, the recognition of these is extremely important"—although more than twenty years have passed since the symptomatology of "chronic benign thyroid insufficiency" or *"myxédéme fruste"* was first described.

This is just as true of the other functional endocrinous disorders, and the fact that they are so very often overlooked, and information as to how to detect them is not easy to obtain, is, it is to be hoped, a sufficiently good reason for this series of articles.

Thyroid insufficiencies are more frequent than the exanthemata. Minor hypothyroidism is among the commonest of disorders. It complicates pediatric problems more often than most other single conditions. It is equally important in the etiology of many functional gynecological troubles as well as in many of the complexi-

---

*E. Hertoghe, "Thyroid Insufficiency," *Practitioner*, January, 1915, page 27.

ties of internal medicine. Neurologists are coming to consider it as a much more vital factor in their difficult cases than has previously been supposed and it may be considered to be an insidious complicating element in many chronic diseases, including that symptom-complex which is usually called "neurasthenia" for the lack of a better name.

Close study is always awarded by results which usually have a very definite clinical significance. This is as true in endocrinology as elsewhere; and to the seeing eye is unfolded many an obscure condition of daily occurrence. These are obscure merely because their insidious onset hides them. They have not been looked for. The taking of the clinical history is too often a perfunctory and unsatisfactory procedure—in general practice, at least—though in the "difficult cases" where others have failed previously, a little more care and thought is given. *The secret of success in endocrinology is thoroughness.* It is "the little foxes that spoil the vines"—the little things that count; and it is surprising how the appreciation of a seemingly insignificant circumstance enables us to correlate some other equally insignificant condition, and thus to pass the unseen barrier which has been separating us from a full understanding of a given case.

The processes of cell-exchange, nutritional and eliminative, influence all parts of the body, hence "no tissue is able to escape the results of impoverishment of the thyroid gland." These results are just as real and important from a practical standpoint as many other obscure but none the less important disorders. They are often even more important, for their very obscurity means that their discovery may be of unusual helpfulness. The fact that they have been overlooked has made a great difference to the treatment, and accounts for many failures; and their discovery may put an entirely new aspect upon the prognosis, for the treatment of internal secretory disorders with well marked and organic imvolvement is not always as successful as that of those conditions in which only the early, functional changes are beginning.

So many organs and symptoms may be affected by disturbed thyroid secretion that it is necessary to consider separately the principal changes in the different tissues. It should be remarked, however, that not all of the conditions shortly to be enumerated will be found together in a given case, not even in the serious forms of hypothyroidism. The detection of a several of them is sufficient ground for the application of suitable thyroid therapy.

This is not empirical thyroid medication, for further proof of its scientific basis is forthcoming when the response is favorable, for Hertoghe's statement must be taken as axiomatic that "those who derive benefit from thyroid medication invariably will be found to show symptoms of thyroid inadequacy"; and if thyroid may have been indiscriminately administered—a not infrequent happening—and the results are favorable, this may be taken as a therapeutic-diagnostic test (just as certain treatment was the only definitive way to demonstrate the etiology of certain brain tumors, a procedure which is now entirely superseded by the Wassermann reaction). The most common example of this is the response of some obese individual to empirical thyroid medication.

As the thyroid plays such an important part in so many of the activities of the body, its functional derangement may result in the commonest of pathological conditions. Inadequate or suppressed thyroid function causes morbid syndromes in direct ratio to the loss of the thyroid hormone to the body; and this may affect almost every tissue and function. Perhaps the most common and constant changes resulting from hyperthyroidism are seen in the skin and its appendages. It is infiltrated with waste products, puffy and insufficiently nourished so that it becomes dry, rough and desquamating. Sensible perspiration is reduced and in advanced cases not even exertion in summer heat will awaken the dormant sweat glands. Usually the more marked edema is only present in myxedema, which will be considered later. Many dermatoses may be found. In children, eczema is most common; at puberty, especially in girls, acne and in adults herpes, psoriasis, urticaria and dermal malnutrition and susceptibility to slight cutaneous infections with varying symptoms. According to Leopold Levi, subthyroidism provides a favorable soil for recurrent erysipelas, and he has found that this condition has yielded readily to thyroid.

The hair is sparse, thin and ill-nourished, falls out easily and characteristic of certain stages of this condition is the *"signe du sourçil"*—a commencing or absolute loss of the outer third of the eyebrows. The nails aren often striated and brittle, later cracking very easily. The teeth are bad, caries being a common result of hyperthyroidism.

The temperature is below normal, and occasionally is lower in the late afternoon, at which time there may be fits of shivering, at times simulating malaria very much. There is a general chilliness and the extremities are cold. These individuals feel the cold very much and require undue amounts of clothing or bed coverings. They are continually complaining about the cold, take all sorts of precaution to guard against it, and slight draughts cause rheumatoid or neuralgic pains. "Dead fingers" are often due to this cause, and cyanosis and even chilblains are connected by many authorities with hypothyroidism. Raynaud's symmetrical gangrene and other forms of vaso-motor spasm with skin manifestations are also credited to the same fundamental cause.

Fatigue, especially in the morning, is usual. Subjects of this disorder were "born tired" and require much sleep. They sleep very heavily, often in the day, especially after eating. There is a feeling of depression and well-marked cases are apathetic, disinterested and "lazy."

The infiltration and generally devitalized condition favors obesity. The muscles, joints and ligaments are all influenced, producing such common symptoms as "rheumatism," stiff neck, aching in the limbs and back often between the scapulae. The joints may be stiff and occasional swelling may even suggest ankylosis. Crackling in the joints is not unusual, and Hertoghe speaks of it being very common in the knees. The involuntary muscles are also affected, the intestinal and abdominal walls are weak and ptosis is the rule. Stasis, constipation and the accompanying toxemia complete a vicious circle. Many of the symptoms so widely emphasized by Sir Arbuthnot Lane were connected and described by Hertoghe fifteen years before and definitely credited to "benign chronic subthyroidism."* For many years he has successfully treated such cases on this basis, and while there may be an advantage in the present vogue of intestinal lubrication and, rarely, even in the operative measures recommended, the elucidation of the fundamental cause and its removal is a much more satisfying as well as rational procedure. Obstipation is not uncommon. The appetite may be progressively poor and certain foods, especially meat, are intensely disliked. The appearance is toxic and often prematurely senile, and in advanced cases a brownish pigmentation of the skin has been remarked.

The heart action is weak and the pulse usually slower than normal. Circulation is especially poor, accounting for some of the manifestations (associated with the infiltration) just connected with hypothermia. Respiratory oppression and varying

---

*E. Hertoghe, *Bull. Acad. of Med. de Belg.*, March, 1899, page 231.

degrees of dyspnea are frequent. Occasionally this is intermittent and mistaken for asthma, thus explaining unexpected cures in "asthma" following thyroid therapy. According to Hertoghe, "the physiologic stimulant of the heart is supplied by the thyroid. It is, in a certain sense, the necessary tonic, the normal digitalis, by which cardiac activity is promoted and maintained. * * * I do not hesitate to exhibit thyroid extract in cases of weak contractility and tendency to syncope, and I may say that the treatment has never failed me."*

The desquamation so marked on the epidermis, as well as the infiltration and loss of function due to it, is also present in the bladder which, by the way, seems to be peculiarly supersensitive to hypothyroidism. Squamous cells are common among the urinary findings, and the incessant denudation results in an undue sensitiveness to contact with the urine, causing frequent urination and enuresis, especially in children who, it will be remembered, are very heavy sleepers (with consequent decreased control over the ejaculator urinae reflex) as a result of which bed-wetting is not unusual. The other urinary findings show the reduced oxidation very plainly. This is especially noticeable in the low urea output. In such cases there is often a tendency to acidosis and an estimation of the ammonia will show that generally it is unduly increased, due to the imperfect metabolism in the liver of the "urea precursors." It almost seems that there is a distinct connection between the thyroid and the liver, for in the more marked cases of hypothyroidism, not only are the chemical functions of the liver disturbed, but it becomes infiltrated, passively congested and tender on pressure. The same is true of the gall bladder, and the desquamation there favors the production of gall stones or jaundice with their usual symptoms.

The mental disturbances are many and varied. Slowness characterizes every form of mental action. The memory becomes gradually poor, there is difficulty in following a line of thought or reasoning. Apathy, somnolence and melancholia, and in the more marked cases organic brain disorders with varying forms of mental deficiency may be present. Headache is a usual and early symptom, especially when early in the day. It is so constant in some cases that they have accustomed themselves to it and "have it all the time." Neuralgia and migraine have been definitely traced to the thyroid, and the conclusions verified by the therapeutic test. Two insidious subjective symptoms may be present

---

*E. Hertoghe, *Practitioner*, January, 1915, page 54.

which are rarely thought of in connection with this disorder. These are giddiness and noises in the ear. The generalized infiltration is again responsible, and the same thing also may cause hoarseness, a change in the *timbre* of the voice, and even aphonia.

The effects of thyroid dyscrasia on the gonads are well marked and among the most constant findings, especially among women. The subject will be more fully considered in the chapter on "Pluriglandular Disorders." There are, however, some important conditions which must be referred to here. Early thyroid disorder spells later reproductive activity. Often the menses are delayed for years, or after having started may be suppressed for a varying period. Amenorrhea is sometimes present, especially in young girls; but when the characteristic infiltration is present the reverse is the rule; menorrhagia, severe and persistent, is a common result. It is believed by some that the thyroid has something to do with the development of the uterus, and that when hypothyroidism is fairly well marked the posterior uterine wall is not properly developed, sometimes causing a marked retroflexion which may be a part of the cause of the menorrhagia. The menses are prolonged, may recommence after they are apparently over, the frequency of the periods is increased and the loss of strength and activity is especially noticeable. "The higher the degree of thyroid inadequacy, the greater the menstrual losses" (Hertoghe).

Many a case of severe dysmenorrhea has an important thyroid element in its causation, and this factor may outweigh all the other associated conditions. These cases nearly always show one or more of the other symptoms of thyroid inadequacy, and the success of thyroid or thyro-luteal therapy* will be the best proof of the correctness of our surmises.

The principal digestive symytoms in addition to those already mentioned are not well marked. Glandular insufficiency (due both to toxemia and infiltration) may be expected, the stools are hard because of the reduced alimentary secretions, and constipa-

---

*My routine in most cases of functional dysmenorrhea is a modification of a method suggested by Professor Dalche, of Paris. It consists of administering ¼ or ½ a grain of thyroid with 5 grains of lucein (corpus luteum) twice a day for the first ten out of twenty days before the menses are due, and four times a day for the ten days prior to menstruation, i. e. doubling the amount in the last ten days. This is repeated for several months, and, later, the medication is given during a shorter period, first a week in which two doses are given daily, and four doses daily for the week before the menses, and still later three days during which the two doses are taken and four doses for the three days before the expected flow.

tion is the rule. I have found emetine very helpful in such cases, and I am sure that when endamebiasis is present—it is much more frequent than we have thought—this is simultaneously benefited. A sensation of heaviness over the epigastrium is not unusual and biliary colic has been caused by infiltration and nothing else; and nutrition is below par, though the weight may be normal or, more often, increased. There is a reduction in weight when our therapeutic efforts begin to relieve the infiltrated cells throughout the body of their accumulated wastes. Hertoghe uses the term "thyroid inanition," which refers to a condition of cell starvation, inactivity and asthenia without obvious changes in contour or weight. Vomiting, in some cases, may be due to this same fundamental cause, especially when associated with pregnancy. (This whole subject will be more thoroughly studied in the chapter on the gonads.)

We have enumerated many symptoms referable to thyroid insufficiency; so many, in fact, that a catalogue of the possible symptoms of this disorder render the cataloguer open to ridicule! The fact remains that they may all occur as a result of this disorder though we may not find more than three or four of these signs in one case. However, it is by no means uncommon to find an individual showing what might be called "a classic picture" of hypothyroidism—severe headaches, neuralgia, "rheumatism," constipation, ptosis, skin disorders, hypothermia, chilliness or distinct chills, slight dyspnea, perhaps better referred to as a sense of undue oppression on the slightest effort, asthenia, mental changes of a minor character as loss of memory, inability to concentrate, menorrhagia, etc.

Such cases, heretofore an unmitigated nuisance both to their relatives and their physician—for too often they have perambulated from one physician's office to another—may now be considered as of unusual profit, for their treatment by attention to the necessary hygienic measures plus thyroid therapy very often means results, the like of which cannot be obtained in any other manner. Such patients are amazed at their progress, their friends see changes in their features and their "view of life" that makes for *success* in practice.

Let us not allow the slightest phase of minor thyroid disorder to pass us again; and let us always remember that *if our diagnosis is wrong the treatment will show it!*

---

Next month: The More Serious Organic Thyroid Diseases." Just as helpful.—Ed.

